# Inhibitory Effects of Human Primary Intervertebral Disc Cells on Human Primary Osteoblasts in a Co-Culture System

**DOI:** 10.3390/ijms19041195

**Published:** 2018-04-13

**Authors:** Rahel D. May, Daniela A. Frauchiger, Christoph E. Albers, Lorin M. Benneker, Sandro Kohl, Benjamin Gantenbein

**Affiliations:** 1 Tissue and Organ Mechanobiology, Institute for Surgical Technology and Biomechanics, University of Bern, CH-3014 Bern, Switzerland; rahel.may@istb.unibe.ch (R.D.M.); daniela.frauchiger@istb.unibe.ch (D.A.F.); 2 Department of Orthopaedic Surgery and Traumatology, Inselspital, Bern University Hospital, University of Bern, CH-3010 Bern, Switzerland; christoph.albers@insel.ch (C.E.A.); lorin.benneker@insel.ch (L.M.B.); sandro.kohl@insel.ch (S.K.)

**Keywords:** human primary intervertebral disc cells, human primary osteoblasts, co-culture, spinal fusion, BMP antagonists

## Abstract

Spinal fusion is a common surgical procedure to address a range of spinal pathologies, like damaged or degenerated discs. After the removal of the intervertebral disc (IVD), a structural spacer is positioned followed by internal fixation, and fusion of the degenerated segment by natural bone growth. Due to their osteoinductive properties, bone morphogenetic proteins (BMP) are applied to promote spinal fusion. Although spinal fusion is successful in most patients, the rates of non-unions after lumbar spine fusion range from 5% to 35%. Clinical observations and recent studies indicate, that the incomplete removal of disc tissue might lead to failure of spinal fusion. Yet, it is still unknown if a secretion of BMP antagonists in intervertebral disc (IVD) cells could be the reason of inhibition in bone formation. In this study, we co-cultured human primary osteoblasts (OB) and IVD cells i.e., nucleus pulposus (NPC), annulus fibrosus (AFC) and cartilaginous endplate cells (CEPC), to test the possible inhibitory effect from IVD cells on OB. Although we could see a trend in lower matrix mineralization in OB co-cultured with IVD cells, results of alkaline phosphatase (ALP) activity and gene expression of major bone genes were inconclusive. However, in NPC, AFC and CEPC beads, an up-regulation of several BMP antagonist genes could be detected. Despite being able to show several indicators for an inhibition of osteoinductive effects due to IVD cells, the reasons for pseudarthrosis after spinal fusion remain unclear.

## 1. Introduction

Chronic low back pain (LBP) is a global disease that has evolved into a socio-economic burden [1,2,3]. LBP has a lifetime prevalence of 65–80% and is often associated with damaged or degenerated intervertebral discs (IVD). If non-conservative treatment options are not successful, like pharmacological or physical therapy, more invasive interventions are required [4]. The current “gold standard” surgery is discectomy followed by spinal fusion. Here, the empty space created by the discectomy is filled with autologous bone graft or non-autologous material like cancellous chips, demineralized bone matrix, ceramics, tricalcium phosphate and hydroxyapatite. Subsequently, the two adjacent vertebral bodies are immobilized with pedicle screws and/or a cage. This fixation should provide optimal conditions for successful osseointegration [5]. Spinal fusion has proven to be an effective procedure in adult patients with prolonged symptoms due to degenerative spine disease, instability, spondylolisthesis and deformity. However, the rates of pseudarthrosis (failure of fusion) after lumbar spine fusions range from 5% to 35% [6,7,8]. Due to the advancement of minimal invasive surgeries, such as laparoscopic anterior spinal fusion, it has become more challenging to remove all the disc tissue sufficiently. However, this is of high importance as clinical observations indicate that partial removal of the disc during discectomy might cause failure in bone formation. This is in accordance with recent findings by Chan et al., which showed that mesenchymal stromal cells (MSC) are hindered to undergo osteogenesis when co-cultured with IVD cells but are stimulated to undergo bone formation with osteogenic medium [9]. Furthermore, Li et al. indicated, in an in vivo pig model, an unsuccessful spinal fusion due to presence of nucleus pulposus cells (NPC) [10].

Two major pathways have particularly strong influences on bone mass or bone metabolism: (i) bone morphogenetic protein (BMP) signaling via SMAD1/5/8 phosphorylation; and (ii) Wnt/β-catenin signaling cascade [11,12]. BMPs are members of the transforming growth factor β (TGF-β) superfamily and play an essential role in skeletal tissue formation by inducing the commitment of MSC towards osteoblasts (OB). Hence, BMPs are involved in bone and cartilage formation in embryonic development, postnatal bone metabolism and fracture healing [13]. Over the last decades, BMPs and BMP antagonists have been investigated to promote fracture healing and bone regeneration. The second well-researched signaling pathway involves Wnt signaling through stabilization of β-catenin translocating into the nucleus and induction of primary cell responses such as proliferation and induction of osteogenesis. Here, antibodies to block sclerostin (SOST) have been primarily investigated in recent decades to target improved bone healing [14]. The role of Wnt signaling has also been shown to be important for cartilage homeostasis [15] and for IVD cells and IVD degeneration [16,17].

Today, BMPs are used in clinics to treat fractured non-unions [18]. Moreover, BMPs and their antagonists have been recently shifted into the focus of developmental biology and very basic science to understand and localize bone formation [19,20,21,22,23]. The BMP signaling pathway is influenced by BMP antagonists, which block BMP signal transduction at multiple levels. Members of BMP antagonists, including noggin (NOG), chordin (CHRD), gremlin 1 and 2 (GREM1 and GREM2), follistatin (FST) and twisted gastrulation BMP signaling modulator 1 (TWSG1), negatively regulate BMP signal transduction by competing with BMP ligands [21,24,25,26]. Hence, the imbalance between the BMPs and their antagonists might be the reason for spinal non-union. Recently, it could be shown that IVD cells express BMP antagonists like NOG, GREM1 and CHRD [9,27]. However, how the IVD cells interact with the OB during osteogenic stimulation is still unknown.

In this study, we investigated the behavior of OB when co-cultured with allogeneic IVD cells. With this co-culture system, we aimed to mimic the postoperative situation of the two cell types, when IVD cells and OB are co-cultured in direct environment, influencing each other. Comparable to the previously observed inhibition of osteogenesis in MSC when they were co-cultured with annulus fibrosus (AFC) and NPC. This study aimed to investigate the osteoinductive effects on OB when co-cultured with the different types of IVD cells, i.e., NPC, AFC and cartilaginous endplate cells (CEPC). For the investigation of our aims we monitored different markers of the three stages of osteoblastogenesis: proliferation, matrix maturation and mineralization [28]. This was achieved by a 21-day experiment followed by investigation of the OB monolayer, quantification of the relative gene expression of the cells and the ossification on both sides. In this study, we observed an effect of human primary IVD cells on bone formation of human primary OB and hypothesize that similar to MSC, also human primary OB show a negative effect on bone formation when co-cultured with human primary IVD cells.

## 2. Results

### 2.1. Matrix Mineralization and Alkaline Phosphatase (ALP) Activity of Human Primary OB

**Figure 1:**
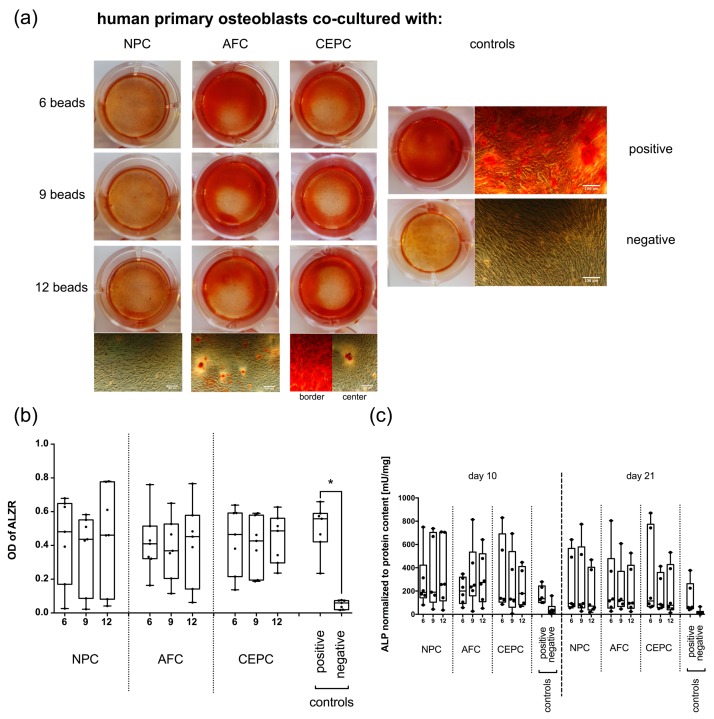
(**a**) Macroscopic and microscopic view of Alizarin red S (ALZR) staining of human primary osteoblasts (OB) co-cultured with 6, 9 or 12 alginate beads of human primary nucleus pulposus cells (NPC), annulus fibrosus cells (AFC), cartilaginous endplate cells (CEPC) or 6 empty beads (positive control) and negative control. All experimental groups were stimulated with osteogenic medium except the negative control, where OB were cultured with basal medium and (**b**) quantification of staining. Data is presented as min to max with median and all data points. Kruskal-Wallis test, * *p*-value < 0.05 *N* = 7. (**c**) Alkaline phosphatase (ALP) activity normalized to protein content was measured at day 10 and 21. Data is presented as min to max with median and all data points *N* = 7.

The positive control (OB stimulated with osteogenic medium, culture insert and empty beads) showed significantly stronger Alizarin red S (ALZR) staining (0.50 ± 0.14, mean ± standard deviation (SD)) compared to negative controls (0.05 ± 0.03, *p*-value < 0.05). In experimental groups, where OB were stimulated with different numbers of IVD beads, a trend of lower calcium deposition could be observed. Especially in the groups of OB stimulated with different numbers of NPC beads, a spread in the intensity of matrix mineralization could be monitored (Figure 1a,b).

Alkaline phosphatase (ALP) activity was measured at days 10 and 21 (Figure 1c). The positive control showed an overall higher ALP activity/total protein content than negative control. Whereas the experimental groups showed a comparable level as the positive control group. However, experimental groups of some donors showed a trend of higher ALP activity/total protein content than the corresponding positive control.

### 2.2. Expression of Major Bone Genes in Human Primary OB

**Figure 2:**
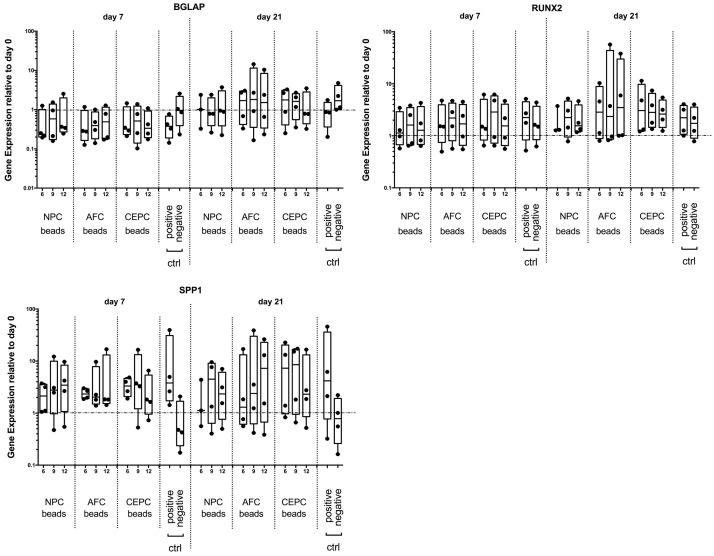
Relative gene expression of major bone genes of human primary osteoblasts (OB) co-cultured with 6, 9 or 12 alginate beads each containing ~80,000 human primary nucleus pulposus cells (NPC), annulus fibrosus cells (AFC), cartilaginous endplate cells (CEPC) or 6 empty beads (positive controls) and negative control. All experimental groups were stimulated with osteogenic medium except negative control, where OB were cultured with basal medium. Gene expression was measured at days 7 and 21. Data are presented as min to max with median and all data points, *N* = 4.

Expression in human primary OB of major bone genes such as osteopontin (*SPP1*), osteocalcin (*BGLAP*) and runt-related transcription factor (*RUNX2*) were measured at days 7 and 21 (Figure 2). SPP1 showed a trend to be down-regulated at days 7 and 21 in the negative control, whereas the positive control showed up-regulation at both time points compared to the day 0 sample. OB in experimental groups showed a trend to be expressed at lower levels at day 7 compared to the positive control, whereas they were up-regulated at day 21. *BGLAP* was down-regulated at day 7, whereas the negative control remained unaffected compared to day 0. At day 21, gene expression changed and OB of the experimental groups showed up-regulation of *BGLAP*, especially in the case of OB co-cultured with AFC. *RUNX2* was up-regulated in OB of the experimental groups, again especially in groups of OB co-cultured with AFC at day 21.

### 2.3. Inhibition of Intracellular Signaling

**Figure 3:**
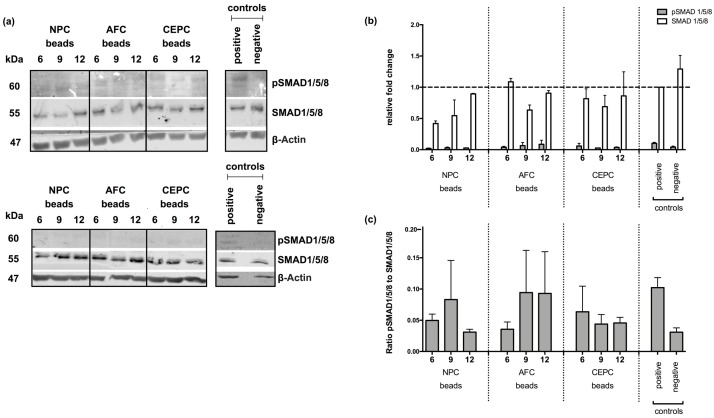
(**a**) Western blot and (**b**) fold change of SMAD and phospho-SMAD 1/5/8 (pSMAD1/5/8) normalized to actin and to positive control (=1.0) (**c**) ratio of pSMAD 1/5/8 and SMAD 1/5/8 of human primary osteoblasts (OB) co-cultured with 6, 9 or 12 alginate beads of human primary nucleus pulposus cells (NPC), annulus fibrosus cells (AFC), cartilaginous endplate cells (CEPC) or 6 empty beads (positive control) and negative control. All experimental groups were stimulated with osteogenic medium except negative control, where OB were cultured with basal medium. Cells were lysed at day 10. Values in (**b**,**c**) are means ± standard error of mean (SEM), *N* = 2.

Protein quantification of phospho-SMAD1/5/8 (pSMAD1/5/8) and SMAD1/5/8 was performed to assess the effect of IVD cells on intracellular BMP signaling (Figure 3a). Relative protein analysis of pSMAD1/5/8 to SMAD 1/5/8 could be assessed at a higher level (0.102 ± 0.016) compared to the negative control (0.041 ± 0.016) and experimental groups OB co-cultured with 6 NPC beads (0.021 ± 0.006), 9 NPC beads (0.030 ± 0.013), 12 NPC beads (0.027 ± 0.004), 6 AFC beads (0.039 ± 0.015), 9 AFC beads (0.065 ± 0.0506), 12 AFC beads (0.087 ± 0.064), 6 CEPC beads (0.058 ± 0.043), 9 CEPC beads (0.027 ± 0.002) and 12 CEPC (0.036 ± 0.010) (Figure 3b). Furthermore, the ratio of pSMAD1/5/8 to total SMAD 1/5/8 was evaluated (Figure 3c). Experimental groups showed a decreased ratio of activated SMAD 1/5/8 compared to the positive control.

### 2.4. Expression of Major IVD/Bone Marker and Several BMP Antagonist Genes in 3D IVD Cell Alginate Culture

The three IVD cells, human primary NPC, AFC and CEPC were investigated for major IVD and bone marker genes, in addition to BMP antagonists after 21 days of cultivation in osteogenic medium and in the presence of OB. All three cell types, human primary NPC, AFC and CEPC showed an up-regulation of IVD marker genes, such as collagen type II α chain 1 (*COL2*) and aggrecan (*ACAN*) compared to day 0 cells. Investigated bone genes showed no major change, except for CEPC, where a significant down-regulation of collagen type I α chain *2* (*COL1*) could be observed (*p*-value < 0.05). Furthermore, a trend of up-regulated *SPP1* and *RUNX2* was observed for CEPC. *TWSG1* was significantly up-regulated in CEPC (*p*-value < 0.05). With the exception of *CHRD*, all other investigated BMP antagonists, i.e., *FST*, *GREM1*, *GREM2*, *NOG* and *TSWG1*, showed a trend to be up-regulated in all three cell types.

## 3. Discussion

Spinal fusion is today’s “gold standard” to treat pain in patients as a result of degenerated or damaged disc. However, despite this current widespread treatment, there are still cases of non-unions, which may lead to pseudarthrosis after lumbar spine surgery. In this study, we aimed to find a model to mimic the postoperative situation of spinal fusion surgery. This was achieved by the co-culture of the three different cell types of the IVD (NPC, AFC and CEPC) with OB. Moreover, human primary cells were investigated in this model, which should best mirror the actual situation in patients.

Chan et al. [9] performed a similar study, where NPC and AFC were co-cultured with human primary MSC. They could successfully demonstrate that matrix mineralization was significantly reduced in MSC when co-cultured for 21 days with NPC or AFC. In the present study, we observed for some donors a trend of lower matrix mineralization compared to the positive control. Especially NPC showed (in two donors) strong inhibitory effects on calcium deposits in OB monolayers. However, the effect seemed to be coherent with different donors (Figure 1 and Figure 2). IVD cells of two donors (i.e., donors 1 and 2, see Table 1) showed strong inhibitory effects on respective OB donors, whereas other OB donors seemed to be resistant to the presence of IVD cells. In the study of Chan et al. [9], ALP activity was measured at day 21, which showed no significant decrease in MSC co-cultured with AFC or NPC. The same effect could be observed in our study. A further difference might be that we normalized ALP activity to total protein content at days 10 and 21. For most of the donors, ALP activity/total protein content had a comparable level to the positive control, whereas the negative control showed a trend to have a lower level. Also here, data were strongly donor-dependent, as in some cases, OB stimulated with IVD cells even showed an increase in ALP activity compared to the positive control. However, Albers et al. [29] showed a decrease in ALP activity with an increasing amount of the BMP antagonist NOG. This is in agreement with our recent study [27] where we could show this same effect by addition of the BMP2 analog L51P, which is known to be a generic antagonist of BMP inhibitors. We could demonstrate a reversal of the inhibiting effects of the secreted factors of IVD cells on the level of histology (ALZR staining) and relative gene expression markers. However, for ALP analysis, we found even a decrease of activity by addition of L51P [27]. A similar effect of reduction of ALP was observed in Albers et al. [29] by addition of the BMP2 analog, L51P. A recent review about the reliability of using ALP as a marker for osteogenesis questioned the cross-comparability across biocompatibility studies [30], where these authors compiled 24 ALP assays across five laboratories.

Besides ALP, which is considered an early marker of the OB differentiation process, other bone markers like *SPP1*, *BGLAP* and *RUNX2* were investigated in human primary OB by qPCR. *SPP1* is twice up-regulated, during proliferation and in the later stages of ossification [28]. This behavior could also be observed in our study. Whereas the positive control showed a trend for up-regulated *SPP1* expression, the negative control did not show an effected in expression level compared to day 0. Experimental groups showed a trend to be up-regulated at day 7 but not to the same extent as the positive control. *BGLAP*, which is considered as a late marker and was described to appear mainly with matrix mineralization [27,28], was also up-regulated in our experiment in a later stage at day 21 but not at day 7. *RUNX2*, which is not only expressed in early osteoprogenitors but is also a key regulator in OB function was up-regulated at days 7 and 21 in all of the experimental groups. Especially OB stimulated with AFC showed the trend of a strong *RUNX2* overexpression at day 21. Whereas total SMAD1/5/8 were not observed to change, experimental groups were not able to induce phosphorylation in SMAD1/5/8, compared to the positive control, where phosphorylated SMAD1/5/8 was indicated. This result indicated that IVD cells might influence the BMP signaling pathway through secretion of BMP antagonists. However, in this study no significant effect between the different groups could be observed, which questions the presence of inhibitors or the lack of receptors for the used OB derived from knee surgery. There is a lack of evidence of dose-dependent effects from an increasing number of IVD cells (shown by qPCR and Western blotting data, Figure 2 and Figure 3). However, Western blot analysis of phosphorylated SMAD 1/5/8 indicated that experimental groups in general revealed a trend towards a lower activation of the TGFβ signaling pathway (Figure 3).

**Figure 4:**
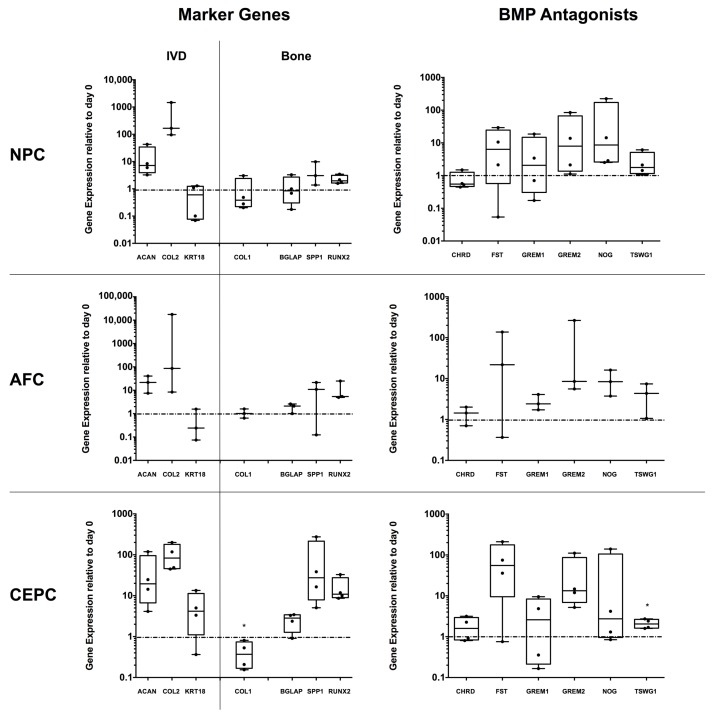
Expression of intervertebral disc and major bone marker and BMP antagonist genes in annulus fibrosus (AFC), nucleus pulposus (NPC) and cartilaginous endplate cells (CEPC) seeded in alginate beads and cultivated for 21 days in co-culture with human primary osteoblasts and stimulated with osteogenic medium. Data are presented as min to max with median and all data points. Gene expression was normalized to day 0 cells of the specific cell type. Significance was tested against day 0 cells. Students *t*-test, * *p*-value < 0.05, *N* = 4.

Besides analysis of osteoinductive effects on the side of OB monolayer we, furthermore, investigated the relative gene expression of NPC, AFC and CEPC in 3D alginate beads, stimulated with osteogenic medium and OB monolayer. Recently, we and others have shown that IVD cells naturally express BMP antagonists such as NOG, GREM1 and CHRD [9,27,31]. We could observe an up-regulation in several BMP antagonists such as *NOG*, *GREM1/2*, *FST* and *TWSG1* in all three cell types, NPC, AFC and CEPC, compared to the respective day 0 cells. For the question whether IVD cells can undergo osteogenesis, we have previously investigated this in a particular IVD-specific progenitor cell population positive for angiopoietin-1 receptor (also known as Tie2) in relatively young bovine IVDs [32]. This trend could now also be observed in NPC, AFC and CEPC (Figure 4) at the osteogenic genes *BGLAP*, *SPP1* and *RUNX2* (which were up-regulated by 10–100 times). This plasticity of IVD cells to ossify has been recently investigated by Brown et al. (in press) [31] who cultured entire IVD sections in the same osteogenic medium as in this study for eight weeks. They found that there was a large donor variation in the ALZR staining indeed, similar to this study. Furthermore, a strong mineralization was detected in the NP region in one young donor, confirming that the plasticity to form bone is present but seems to be prevented in most patients, possibly by BMP antagonists. A similar trend was observed when BMP2 was injected into the AF region of rabbit IVDs in organ culture [33], where it was found that mainly the outer AF tissue has the capacity to form bone.

In the past, several studies have shown the acceleration in osteoblastic differentiation by using siRNA to knock down NOG [34,35,36,37]. Importantly, all of these studies discussed here were performed in mice or with an OB cell line in contrast to our study, which was conducted with clinically relevant human primary cells. This is the first study to report effects of human IVD cells on human OB in an indirect co-culture to test for secreted factors. The fact that higher *NOG* expression was detected in IVD cells could be that *NOG* has a specific function in the IVD space and plays a pivotal role in the prevention of nerve ingrowth and blood vessel formation [38]. Studies on human primary cells are rather rare but are highly clinically relevant since patient-derived cells are investigated. However, it also offers the problem of high donor variance, as patients are in most cases rather old in age and in different states of health. This variance was reflected in our study especially in matrix mineralization and ALP activity of different donors. Furthermore, OBs were isolated from the femur of patients undergoing total knee replacement. Although an appropriate choice of source for a simple in vitro model to study non-union, in reality, OBs from human femur would not be used to support spinal fusion.

## 4. Materials and Methods

### 4.1. IVD and OB Donor Materials and Cell Isolation

Human IVD tissue was obtained from eight patients ranging from 23 to 61 years of age (mean ± standard error of mean (SEM)) (48.43 ± 5.61 years) undergoing spinal surgery after experiencing trauma to their discs. Immediately after harvesting the IVDs, an experienced surgeon divided the tissues into annulus fibrosus (AF), nucleus pulposus (NP) and cartilaginous endplate (CEP). The tissues were subsequently processed in the laboratory within 24 h after surgery. The human OB were obtained from eight patients undergoing total knee replacement ranging from 24 to 88 years of age (65.0 ± 7.48 years). Patients provided written consent of the patients was obtained and the ethics committee of the Canton of Bern approved the procedure.

The human IVD and OB samples are listed in Table 1. The human disc cells were isolated from their native extra cellular matrix by sequential digestion of the tissues with 1.9 mg/mL pronase (Roche, Basel, Switzerland) for one hour and (NP: 64 U/mL, AF: 129 U/mL, CEP: 1562 U/mL/g per gram CEP) collagenase type 2 (Worthington, London, UK) on a plate shaker at 37 °C overnight. Remaining tissue fragments were removed by using filtration through a 100 μm cell strainer (Falcon, Becton Dickinson, Allschwil, Switzerland). NPC, AFC and CEPC were expanded separately in the proliferation medium, low-glucose (1 g/L) Dulbecco’s Modified Eagle Medium (LG-DMEM, Gibco, Life Technologies, Zug, Switzerland), supplemented with 10% fetal bovine serum (FBS) and penicillin/streptomycin (P/S, 100 μg/mL and 100 IU/mL, respectively, Merck, Darmstadt, Germany) up to passage two, prior to experiments.

The bone tissue was transferred with minimal delay to laboratory. After removing the soft tissue, the cancellous bone was cut into pieces of 3–5 mm in diameter. Then, the pieces were washed with PBS until no remaining hematopoietic marrow was visible. The washed bone fragments were cultured at a density of 0.2–0.6 g of tissue per 75 cm^2^ flask in 10 mL of LG-DMEM supplemented with 10% FBS, P/S and 50 µg/mL l-ascorbic acid-2-phosphate (Sigma-Aldrich, St. Louis, MS, USA). The cultures were then left undisturbed for at least seven days [39]. After the cells reached confluency, the bone fragments were removed and the cells were trypsinized as described above.

### 4.2. Cell Encapsulation and Co-Culture

**Figure 5:**
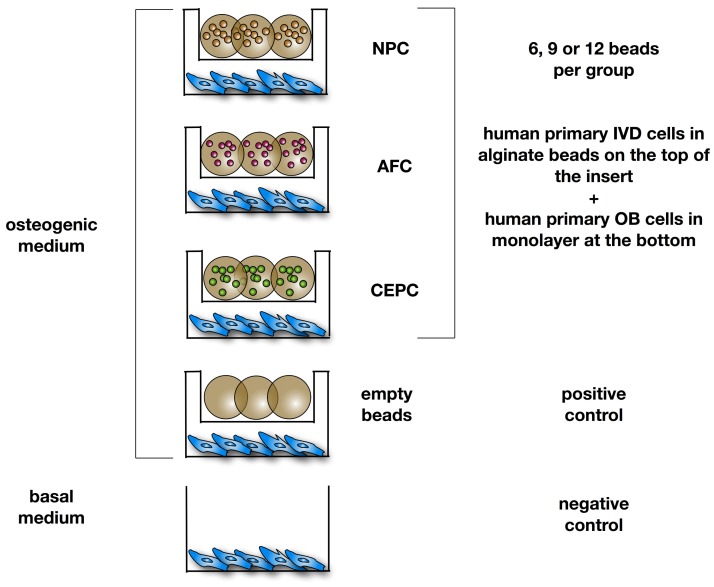
Experimental design of co-culture experiments of primary osteoblasts (OB) with either nucleus pulposus cells (NPC), annulus fibrosus cells (AFC), cartilaginous endplate cells (CEPC) or 6 empty beads as positive control. OB were cultured as monolayer at the bottom of the well, intervertebral disc (IVD) cells were seeded in 3D alginate beads (~30 µL) on the top in the insert with 0.4 µm pore size. All experimental groups and positive control were stimulated with osteogenic medium (α-MEM supplemented with 10% FBS, 100 μg/mL penicillin/streptomycin, 50 µg/mL l-ascorbic acid-2-phosphate, 10 nM dexamethasone and 5 mM β-glycerophosphate). Negative control was stimulated with basal medium (α-MEM + 10% FBS).

OB were seeded at a density of 3 × 10^4^ cells/well in 12-well plates (12-well companion plate, Corning, Kaiserslautern, Germany). NPC, AFC and CEPC were encapsulated separately in 1.2% alginate at a density of 4 Mio/mL by using a syringe (22 G needle). Cell-alginate suspension was then dropped in 102 mM CaCl_2_ solution [40,41]. The alginate beads from all three cell types were distributed in different quantities (6, 9 or 12 beads) in culture inserts (0.4 µm pore size, high pore density, polyethylene terephthalate track-etched, Becton, Dickinson and Company, Allschwil, Switzerland) and co-cultured with the OB monolayer. The experiment included a positive control with OB and culture insert containing empty beads only. The experimental groups and the positive control group were cultured in α-MEM (Gibco) supplemented with 10% FBS, P/S, 50 µg/mL l-ascorbic acid-2-phosphate, 10 nM dexamethasone and 5 mM β-glycerophosphate (all purchased from Sigma-Aldrich). The negative control group included OB cultured without culture inserts and beads with α-MEM without any osteogenic supplements (Figure 5). The experiment was run for 21 days under hypoxic conditions. The medium was changed every second to third day.

### 4.3. Histological Staining of Cell Mineralization

Cell mineralization of OBs was monitored after 21 days by staining calcium deposition with 2% ALZR solution (Sigma-Aldrich). Prior staining, cells were fixed with 4% of formalin for 10 min. The fixed cells were incubated with the ALZR dye for 45 min and then washed three times with distilled water and once with phosphate-buffered saline (PBS, Sigma-Aldrich, Buchs, Switzerland). Images were taken with a digital camera (Eclipse 800, Nikon, Tokyo, Japan).

To quantify the ALZR staining, each well was incubated with 10% cetylpyridinium chloride in 10 mM NaPO_4_ for one hour to release the dye from the fixed cells. Supernatant was measured at 570 nm using a microplate reader (SpectraMax M5, Bucher Biotec inc., Basel, Switzerland).

### 4.4. Alkaline Phosphatase Activity and Total Protein Content

The alkaline phosphate (ALP) activity of OB from all experimental and control groups was measured at days 10 and 21 of the experiment by using an Alkaline Phosphatase, Diethanolamine Detection Kit (Sigma-Aldrich). Prior measuring the ALP activity cells were lysed with CelLytic M (Sigma-Aldrich) and sonicated for 15 s. The assay uses *p*-nitrophenyl phosphate as substrate. The *p*-nitrophenol product formed by hydrolysis was measured after 30 min of incubation at 37 °C at 405 nm using a microplate reader (Bucher Biotec inc.).

To normalize the ALP, the total protein content of each sample was quantified by Bradford assay. Total protein content was determined by using Coomassie blue reagent (Serva GmbH, Heidelberg, Germany) and a Bovine Serum Albumin (BSA) standard (Sigma-Aldrich, Buchs, Switzerland).

### 4.5. Analysis of Specific Gene Expression in Human Primary OB Monolayer and Human Primary IVD 3D Culture with Quantitative Polymerase Chain Reaction (qPCR)

Prior to RNA extraction IVD beads were snap frozen with liquid nitrogen. For each donor three beads taken at day 0 (unstimulated) and at day 21 (after stimulation with osteogenic medium) were used. Alginate beads were pulverized by using liquid N_2_-precooled mortar and pestle and then suspended in TRI reagent (Molecular Research Center, Cincinnati, OH, USA). OB monolayer was lysed directly in TRI reagent. Total RNA was extracted using a modified TRI spin method as reported previously [42,43]. RNA was mixed with polyacryl carrier (Molecular Research Center) and organic 1-bromo-3-chloropropane (BCP, Sigma-Aldrich). With a centrifugation step at 1200× *g* for 15 min at 4 °C the total RNA was separated from the DNA/protein fraction. The clear RNA supernatant fraction was loaded at a final concentration of 70% ethanol onto the GenElute™ Miniprep Kit (Sigma-Aldrich). The digestion of the remaining DNA was performed by using DNase I (AMP-D1, Sigma-Aldrich) for 15 min. RNA integrity and purity was checked on selected samples by Experion™ Automated Electrophoresis System (Bio-Rad, Reinach, Switzerland). For reverse-transcription, the reverse transcriptase “all-in-one cDNA Synthesis SuperMix” was used from Bimake.com (distributed by LuBioScience GmbH, Lucerne, Switzerland) by using ~500 ng of total RNA per 20 µL reaction. cDNA was then diluted 1:4 in Tris-EDTA (TE) buffer prior qPCR, genomic template and negative controls were run to exclude DNA contamination. Real-time PCR was performed in duplicates using SYBR Green PCR mastermix on a CFX96touch RT-qPCR system (all from Bio-Rad). The following bone marker genes were monitored for OB and IVD cells: Runt-related transcription factor 2 (*RUNX2*), bone gamma-carboxyglutamate protein (*BGLAP*, also known as osteocalcin *OCN*), secreted phosphoprotein 1 (*SPP1*, also known as osteopontin *OPN*) (Table 2). The 18S ribosomal RNA gene (*18S*) and glycerine-aldehyde-3-phosphate dehydrogenase (*GAPDH*) genes were chosen as reference genes. Additionally, the IVD cells were investigated for IVD marker genes like *COL2*, *COL1*, *ACAN* [44]. Furthermore, the relative gene expression of different BMP antagonists i.e., *NOG*, *GREM1*, *GREM2*, *FST* and *TWSG1* was measured in IVD cells in 3D culture in alginate beads. All primers were synthesized by Microsynth inc. (Balgach, Switzerland) and were tested for efficiency. The qPCR was run using a two-step protocol with an annealing temperature of 61 °C for 40 s and 95 °C for 15 s and 45 cycles. As a control for the specificity of the amplicons a melting curve analysis was performed after cycling. For the quantification of relative gene expression, the CFX-96 cycler software (Bio-Rad) was used. The number of PCR cycles needed for each sample to reach that threshold level was recorded as the *C*q value [45]. ∆∆*C*q values at end point were estimated relative to day 0 and transformed into relative mRNA values using the formula 2^−∆∆*C*q^ [46] and using two reference genes.

### 4.6. SMAD1/5/8 Protein Quantification by Western Blot Analysis

After 10 and 21 days of co-culture the cells were washed with Tris-buffered saline (TBS) and incubated in CelLytic M (Sigma-Aldrich) for 15 min to lyse the OB monolayer. A phosphatase inhibitor cocktail (Bimake.com) was added to each sample. Then Bradford assay was performed as described above to determine the absolute amount of protein in each sample. 30 µg protein per sample was heated with Laemmli buffer (Bio-Rad) at 95 °C for 5 min. Samples were then subjected to 7.5% sodium dodecyl sulfate polyacrylamide gel electrophoresis (SDS-PAGE) and transferred to a 0.45 µm immobilon-p PVDF membrane (Merck-Millipore, Coisins, Switzerland). Membranes were blocked for unspecific antibody binding by immersing for one hour in 5% BSA (Sigma-Aldrich) in TBS with Tween20 (Sigma-Aldrich) (TBS-T). Membranes were washed several times with TBS-T and primary antibodies were then incubated overnight at 4 °C. As primary antibodies anti-phospho-SMAD1/5/8 (Cell Signaling Technology, Danvers, MA, USA, Cat no. 13825) and anti-SMAD1/5/8 (Santa Cruz Biotechnology, Inc., La Jolla, CA, USA Cat no. sc-6031-R) were used. IRDye 800 CW Goat anti-Rabbit IgG served as secondary antibody, which was incubated for one hour. The membranes were analyzed by using Li-Cor Odyssey Infrared Imaging System (distributed by DMP Inc., Fehraltorf, Switzerland). Protein bands were evaluated semi-quantitatively by using ImageJ (ImageJ 1.48 v, Wayne Rasband, National Institutes of Health, Bethesda, MD, USA).

### 4.7. Statistics

Statistical analysis was performed by using Prism 6.0 h for Mac OS X (GraphPad, La Jolla, CA, USA). Shapiro-Wilk normality test was performed to test for parametric distribution. Kruskal-Wallis was used to test statistical significance. Values are given as means ± SEM. A *p*-value < 0.05 was considered to be significant.

## 5. Conclusions

Co-culture of human, allogeneic, primary IVD cells, i.e., NPC, AFC and CEPC, in 3D with human adult primary OB in monolayer culture under osteogenic conditions did not show significant inhibiting effects.

- Dose-dependent inhibitory effects of IVD cell number on primary OB could not be statistically confirmed at the RNA and protein levels.
- A strong donor variation was found in the clinically derived cells for the inhibition of OBs.

## Figures and Tables

**Table 1:** Donor list of osteoblasts (OB) and intervertebral disc cells (nucleus pulposus (NPC), annulus fibrosus (AFC) and cartilaginous endplate cells (CEPC)). Female = F, Male = M.

No.	OBs			NPC/AFC/CEPC			Pfirrmann Grade	Comments
	Passage	Age	Sex	Passage	Age	Sex		
1	1	65	F	1	51	F	3	Monotrauma, surgery 10 days post injury, osteoporotic patient
2	1	66	F	1	56	M	2–3	Polytrauma, surgery 8 days post injury, healthy athlete
3	1	76	F	2	32	M	1	Polytrauma, surgery 9 days post injury, healthy patient
4	1	88	F	2	57	F	3	Monotrauma, surgery 3 days post injury, injury of endplates, healthy patient
5	1	67	M	2	23	M	1–2	Monotrauma, surgery 2 days post injury
6	1	24	F	2	61	M	3	No trauma, connection instability
7	1	69	M	2	59	M	2	Polytrauma, surgery 11 days post injury, injury of endplates, healthy athlete

**Table 2:** List of genes tested during qPCR of human primary osteoblasts and human primary intervertebral disc cells.

Name	Description	Accession No.	Primer forward	Primer reverse
*18S*	Ribosomal 18S RNA gene	NR_145820.1	CGA TGC GGC GGC GTT ATT C	TCT GTC AAT CCT GTC CGT GTCC
*ACAN*	Aggrecan	XM_017021987.1	AAG GCT GCT ATG GAG ACA A	ACT CAT TGG CTG CTT CCT
*BGLAP (OCN)*	Osteocalcin	NM_199173.5	GCA GAG TCC AGCAAA GGT G	CCA GCC ATT GATACA GGT AGC
*COL1A2*	Collagen type I α2 chain	NM_000089.3	GTG GCA GTG ATG GAA GTG	CAC CAG TAA GGC CGT TTG
*COL2A1*	Collagen type II α1 chain	XM_017018831.1	AGC AGC AAG AGC AAG GAG AA	GTA GGA AGG TCA TCT GGA
*CHRD*	Chordin	XM_017007394.1	GCC TCC GCT TCT CTA TCT	AAC AGG ACA CTG CCA TTG
*FST*	Follistatin	NM_006350.3	GGA CCA GAC CAA TAA TGC	CTC ATA GGC TAA TCC AAT AGA T
*GAPDH*	Glyceraldehyde-3-phosphate dehydrogenase	NM_001289745.2	ATC TTC CAG GAGCGA GAT	GGA GGC ATT GCTGAT GAT
*GREM1*	Gremlin1, DAN family BMP antagonist	NM_001191322.1	GAG AAG ACG ACG AGA GTA AGG AA	CCA ACC AGT AGC AGA TGA ACA G
*GREM2*	Gremlin2, DAN family BMP antagonist	XM_011544249.2	CCA TCC TCA ACC GCT TCT	GAC TCC TCC TCC TTC TTC AC
*KRT18*	Cyto-Keratin 18	NM_199187.1	TCT TGC TGC TGA TGA CTT	CCT CTT CGT GGT TCT TCT
*RUNX2*	Runt-related transcription factor 2	NM_001024630	AGC AGC ACT CCATAT CTC T	TTC CAT CAG CGTCAA CAC
*SPP1 (OPN)*	Osteopontin	NM_001251830.1	ACG CCG ACC AAGGAA AAC TC	GTC CAT AAA CCACAC TAT CAC CTC G
*TSWG1*	Twisted gastrulation BMP signaling modulator 1	NM_020648.5	CCA GCC ACA CCA CCA GAA	ACT CGC AGC AGG CAT TAT GA

## Abbreviations

ACAN	Aggrecan
AF	Annulus fibrosus
AFC	Annulus fibrosus cells
ALP	Alkaline phosphatase
ALZR	Alizarin red S
BGLAP	Osteocalcin
BMP	Bone morphogenetic protein
CEP	Cartilaginous endplate
CEPC	Cartilaginous endplate cells
CHRD	Chordin
COL1	Collagen type 1
COL2	Collagen type 2
FBS	Fetal bovine serum
FST	Follistatin
GREM1	Gremlin 1
GREM2	Gremlin 2
IVD	Intervertebral disc
KRT18	Cyto-keratin 18
LG-DMEM	Low glucose-Dulbecco’s Modified Eagle Medium
MSC	Mesenchymal stromal cells
NP	Nucleus pulposus
NPC	Nucleus pulposus cells
OB	Osteoblast
P/S	Penicillin/streptomycin
qPCR	Quantitative polymerase chain reaction
RUNX2	Runt-related transcription factor 2
SD	Standard deviation
SEM	Standard error of mean
SPP1	Osteopontin
TWSG1	Twisted gastrulation BMP signaling modulator 1
